# The Microbial Profile and Resistance Pattern of Pathogens Isolated From Long COVID Pneumonia Patients and Their Correlation to Clinical Outcome: Our Experience From a Tertiary Care Hospital

**DOI:** 10.7759/cureus.23644

**Published:** 2022-03-30

**Authors:** C Mohan Rao, Pragyan Rout, Ashwini P Pattnaik, Nipa Singh, Aarthi Rajendran, Shubhransu Patro

**Affiliations:** 1 Pulmonary Medicine Deparment, Kalinga Institute of Medical Sciences, Bhubaneswar, IND; 2 Pulmonary Medicine Department, Kalinga Institute of Medical Sciences, Bhubneswar, IND; 3 Nephrology Department, Kalinga Institute of Medical Sciences, Bhubaneswar, IND; 4 Microbiology Department, Kalinga Institute of Medical Sciences, Bhubaneswar, IND; 5 Pulmonary Medicine Department, Kalinga Institute of Medical Sciences, Bhubaneswar, IND; 6 General Medicine Department, Kalinga Institute of Medical Sciences, Bhubaneswar, IND

**Keywords:** microbial profile, pdr, xdr, mdr, long covid

## Abstract

Background

Coronavirus disease 2019 (COVID-19) patients with persistent symptoms for at least four weeks in spite of being reverse transcriptase-polymerase chain reaction (RTPCR) negative for COVID infection are defined as long COVID (wherein pulmonary involvement is seen in a significant proportion of cases). The history of prolonged use of corticosteroids, broad-spectrum antibiotics, and associated comorbid conditions in these patients increases the possibility of infection with multidrug-resistant microbial strains. It may lead to a grave prognosis, hence appropriate microbiological evaluation and management at the earliest can have a better outcome.

Methods

A retrospective observational study was carried out among long COVID patients admitted to the Kalinga Institute of Medical Sciences, Bhubaneswar, Odisha, India, a tertiary care hospital. Eighty-four patients admitted to the ICU or non-ICU ward in the hospital from April to October 2021 were included in the study. Antibiotics, as prescribed by our hospital antibiotic policy, were administered wherever required and were subsequently changed according to culture and sensitivity reports of the samples (sputum, endotracheal aspirates, or blood). An analysis of the antibiotic sensitivity patterns of the pathogens isolated was performed. The outcome after optimum medical management was assessed for survivors, discharge, or death.

Results

Out of the total of 84 patients, 41 samples (sputum, endotracheal aspirates or blood) were collected and sent for culture, of which 32 (78.1%) were found to be culture positive for pathogens. Among the pathogens isolated, there were 22 (69%) drug-resistant and 10 (31%) sensitive organisms. Among the 22 resistant pathogen isolates, 18 were Gram-negative species, the most common species being *Klebsiella pneumoniae, Pseudomonas aeruginosa,* and *Acinetobacter baumanii;* two were Gram-positive species, one each from *Staphylococcus aureus* and *Enterococcus faecalis,* and three were *Candida tropicalis*. Of five deaths reported among 22 cases with resistant isolates, extensively drug-resistant (XDR), multi-drug resistance (MDR), and pan drug resistance (PDR) strains were detected in three, one, and one cases, respectively, and were harboured by *K. pneumoniae, P. aeruginosa,* and *A. baumanii*. Of the total eight deaths, there were two deaths among the 43 patients who received an empiric antibiotic in the wards, and six deaths were reported in the ICU. Despite raised biomarkers of inflammation, comorbid illnesses, renal impairment, and immunocompromised states, there was 91% survival and discharge, which was statistically significant (p-value = 0.00).

Conclusion

To conclude, *K. pneumoniae, P. aeruginosa, A. baumanii, C. tropicalis, S. aureus, and E. faecalis *were the most commonly isolated organisms among long COVID pneumonia cases, of which some were MDR, PDR and XDR strains. Early microbiological evaluation with targeted, proper antimicrobial usage along with optimized medical management and, wherever needed, critical care support in the ICU may lead to a better prognostic outcome in those groups of patients.

## Introduction

After a brief decline in the Coronavirus disease 2019 (COVID-19) pandemic, there was an increase in the admission of COVID-19 cases from April 2021 onwards, mostly caused by the Delta variant of the SARS-CoV-2 virus, which hit India badly. Its exponential spread in a dense population was facilitated due to its highly transmissible nature. Clinically, it also manifests in severe form, resulting in higher morbidity and mortality in India.

The delta variant infected 4.26 crores of people, out of which 5.08 lakhs have succumbed to the illness [1]. As the cohort of survivors expands, the lingering after-effects of the illness are experienced by the patients with the defining syndrome called long COVID, which has new or persistent symptoms for four or more weeks. This long-COVID syndrome comprises multisystem involvement, including the pulmonary, cardiovascular, central nervous system, neuropsychiatric, and gastrointestinal systems [2].

Pulmonary involvement constitutes an important component of target organ involvement that manifests symptoms like breathing difficulty, fever, chest pain, and persistent dry cough. These symptoms need proper treatment after categorization of presentation as pneumonia, obstructive airway disease, or interstitial lung disease in order to attain an optimum health-related quality of life. Pharmacotherapy of these respiratory illnesses is not sufficient to provide the optimum cure [3]. These groups of cases suffer from hypoxia, exercise limitation, and persistent cough that leads to stress on the health care system. During the admission to COVID hospital, these cases were administered with broad-spectrum antibiotics as per our institute's infection control guidelines.

However, the study on the involvement of pathogens/drug-resistant pathogens in long COVID is limited. Thus, the present study aims to unravel the causative pathogens, their sensitivity/resistance patterns, and their impact on the outcome of long COVID presentations.

## Materials and methods

A retrospective observational study was conducted at the post-COVID ward in a tertiary health care center during the period from April to October 2021 with the approval of the Institutional Ethics Committee (IEC No. KIIT/KIMS/IEC/764/2021).

A total of 84 admitted patients were included in the study, with the inclusion criteria being those cases with an age of ≥18 years who remained symptomatic for at least four weeks after the diagnosis of COVID-19, although the reverse transcriptase-polymerase chain reaction (RTPCR) result was negative. The exclusion criteria were those patients with an active or previous history of pulmonary tuberculosis, age less than 18 years, and pregnancy.

The important features of patients such as age, sex, clinical features, duration of hospitalization, co-morbidities, the severity of illness at the time of diagnosis, CT thorax involvement, liver and renal biochemistry results, complete blood count, the requirement of mechanical ventilation, microbes isolated from sputum or endotracheal aspirates, and/or blood culture samples with sensitivity and resistance results were retrieved, analyzed, and correlated retrospectively with the clinical outcome in terms of duration of hospitalization, morbidity, and mortality. Microbiological profiles and antibiotic sensitivity of isolates from clinical samples were performed in our microbiology laboratory as per protocol [4-6].

From the record, we observed that samples (blood, endotracheal aspirate, and sputum) from 41 patients were sent for analysis. The respiratory samples (endotracheal aspirate and sputum) were processed as per standard microbiological procedures, and for blood, the BACTEC instrument was used. Subsequently, identification and antibiotic sensitivity were determined as per CLSI in the Vitek 2 compact instrument.

Culture positivity was seen in 31 samples and its clinical outcome was adjudged as per the survival and mortality of the patients. The correlation between clinical outcome and its resistance pattern was made as follows: (i) multi-drug resistance (acquired resistance to at least one agent in three or more antimicrobial categories), (ii) extensive drug resistance (bacterial isolate susceptible to only one or two antimicrobial agents), (iii) pan drug resistance (non-susceptibility to all antibacterial categories). The resistance pattern has been determined as per the guidelines of the European Center for Disease Control (ECDC) and the Center for Disease Control, Atlanta (CDC) [7].

The patients included in the study were grouped into three categories based on their clinical severity at presentation, intensive care unit (ICU) requirement, and need for mechanical ventilation.

Data were analyzed using SPSS 16.0 software (IBM Corp., Armonk, NY), Fisher's exact test, and Pearson's chi-squared test. The association between variables was deemed not significant (NS), significant (S), and highly significant (HS) when the p-value was >0.05, <0.05, and <0.001, respectively.

## Results

Demographic and clinical features of long COVID

Of the total patients, 22 were admitted to the ICU based on clinical evidence of respiratory distress and were on invasive mechanical ventilation. Eighteen were on non-invasive ventilation. Forty-four patients were in wards (non-ICU) for appropriate medical therapy.

Demographic features showed that the number of male patients was higher compared to females (2.65:1). The ratio of males to females in subgroups of patients in the ICU on an invasive mechanical ventilator, in the ICU on oxygen (non-invasive ventilation), and in the ward was 1:1, 3.5:1, and 4.5:1, respectively (Table 1). The mean age of patients in the critically ill, severely affected, and stable patient groups was 58.8 ± 13.13, 53.3 ± 23.3, and 50.3 ± 9.13 years, respectively (p=0.078). Analysis of the clinical features showed that irrespective of the disease severity, the most common presentation among long-COVID patients was dyspnea (71.42%), followed by cough (41.67%) and fever (42.86%).

**Table 1:** Demographic and clinical features of long COVID patients based on the severity ICU: intensive care unit, CRP: C-reactive protein, TLC: total leukocyte count, LFT: liver function test, NA: not applicable, NS: not significant, S: significant, HS: highly significant

Variable	ICU on invasive mechanical ventilator (%) N = 22	ICU on oxygen (Severe) (%) N=18	Ward patients (%) N = 44	Total (%)	P-value (p-value < 0.05)
Demographic profile					
Mean age (in years)	58.8 ±13.13	53.3±23.3	50.3±9.13	NA	NS (0.078)
Male	11(50)	14(77.7)	36(81.82)	61(72.62)	S (0.02)
Female	11(50)	4(22.22)	8(18.18)	23(27.38)	
Clinical features					
Cough	10(45.45)	9(50)	16(36.36)	35(41.67)	NS (0.56)
Dyspnea	20(90.91)	16(88.89)	24(54.54)	60(71.42)	HS (0.00)
Chest pain	2(9.09)	4(22.22)	11(25)	17(20.24)	NS (0.30)
Fever	12(54.6)	12(66.67)	12(27.27)	36(42.86)	NS (0.11)
Fatigue	3(13.64)	1(5.56)	5(11.36)	9(10.71)	NS (0.11)
Co-morbidities					
Diabetes mellitus	8(36.36)	3(16.67)	16(34.09)	26(30.95)	NS (0.32)
Hypertension	11(50)	7(38.8)	16(36.36)	34(40.48)	NS (0.56)
Lab parameters					
Median of serum procalcitonin (in ng/ml)	1.97	0.20	0.11	NA	S (0.02)
Median of CRP (mg/L)	120	76.93	29.97	NA	S (0.02)
Median of D-dimer (g/L)	2.9	1.05	0.75	NA	NS (0.11)
Median of TLC	13750	11210	8910	NA	S (0.03)
Deranged LFT	8(36.36)	3(16.67)	9(20.45)	20(23.81)	NS (0.15)
Deranged renal function	13(59.09)	6(33.33)	8(18.18)	27(32.14)	HS (0.004)
Radiological parameters					
Ground glass opacity	5(22.73)	3(16.67)	14(31.8)	22(26.19)	NS (0.72)
Consolidation	15(68.2)	13(72.2)	22(50)	50(59.52)	
Interstitial septal thickening	2(9.09)	1(5.56)	4(9.09)	7(8.33)	
Pleural effusion	0	1(5.56)	3(6.68)	4(4.7)	
Pneumothorax	1(4.54)	0	0	1(1.19)	
Clinical course and outcome					
Non-invasive ventilation requirement	0	18(100)	0	18(21.4)	NA
Invasive mechanical ventilation requirement	22(100)	0	0	22(26.19)	
The median length of stay (in days)	13.5	19	8	NA	S (0.01)
Death	6(36.36)	0	2	8(9.52)	HS (0.000)

Diabetes and hypertension constitute the major share of co-morbidities among the long-COVID cases. C-reactive protein was elevated in 21 (95%) cases among the critically ill patients and all (100%) in the severe category, compared to 28 patients (63.7%) among those admitted, with values being 120 mg/L, 76.8 mg/L, and 29.9 mg/L, respectively (p=0.02).

Similarly, median D-dimer values were 2.9 g/L, 1.05 g/L, and 0.75 g/L among 18 (82%) in the critical category, 14 (78%) in the severe category, and 17 (39%) in ward patients, respectively (p = 0.11).

The median total leukocyte count was higher in the critically ill as compared to the severe and ward patient categories (p=0.03). Deranged liver and renal function tests were seen in 59% of critically ill patients, 33.3% of severe category patients, and 18.18% of ward patients (p=0.004). Serum procalcitonin was elevated in critically ill patients with a median value of 1.97 ng/ml while in severely affected and ward patients it was 0.2 ng/ml and 0.1 ng/ml, respectively (p=0.02). Consolidation was the predominant radiologic finding among critically ill (68.2%), severely ill (72.7%), and ward (50%) patients, respectively (p=0.72).

The median duration of hospitalization was 13.5 days for the critically ill, 19 days for the severely ill, and 8 days for ward patients (p=0.01). Out of the eight deaths, six occurred among critically ill patients, two among ward patients, and no mortality was observed in severely affected patients (p=0.00).

Microbiological profiling and the antibiotic sensitivity/resistance pattern of isolates from clinical samples

Of the 84 patients, 41 samples (sputum, endotracheal aspirate, and blood) were collected from different types of long COVID patients and were cultured for the detection of pathogens. Pathogens were detected in 31 samples, with 32 isolates. Among 32 isolates, most of the microbes were detected in sputum, endotracheal aspirate (71.8%), and the rest in blood samples (28.2%). More isolates (51%) were detected in critically ill patients than in non-ICU ward patients (25%) (Table 2).

**Table 2:** Site-specific etiological distribution of pathogens. *Same isolate has been obtained from two samples of the same patient.

Organisms	Samples from which isolated		
	Endotracheal secretion	Sputum	Blood
Klebsiella pneumoniae	1	8	1
Escherichia coli	0	1	1
Proteus mirabilis	1 *	0	1*
Morganella morganii	0	0	1
Pseudomonas aeruginosa	2	3	1
Burkholderia cepacia	0	1	0
Acinetobacter baumanii	0	4	1
Staphylococcus aureus	0	0	1
Enterococci faecalis	0	0	1
Candida tropicalis	1 + 1*(2)	0	1 + 1*(2)
Total no. of isolates	6	17	10

Isolates were mostly detected from sputum in 17 (53.1%), followed by endotracheal aspirate 6 (18.7%) and blood 10 (28.02%), bringing the overall success of pulmonary sampling to 23 (71.8%) (Table 3).

**Table 3:** Detection of pathogens in clinical sample of different type of long COVID cases. Pathogens detected in different groups of patients such as critically ill (patient with mechanical ventilation support), severe category (Patient with oxygen support) and general ward (stable group).

Type of patients	Samples with no microbial growth (n = 10)	Samples which showed growth (n = 31)	Total samples cultured (n = 41)	P-value
Critical ill	3(30%)	16(51%)	19	HS(0.003)
Severe category	2(20%)	7(22.58%)	9	
General ward	5(50%)	8(25.80%)	13	

Resistance pattern of the culture isolates

**Figure 1:**
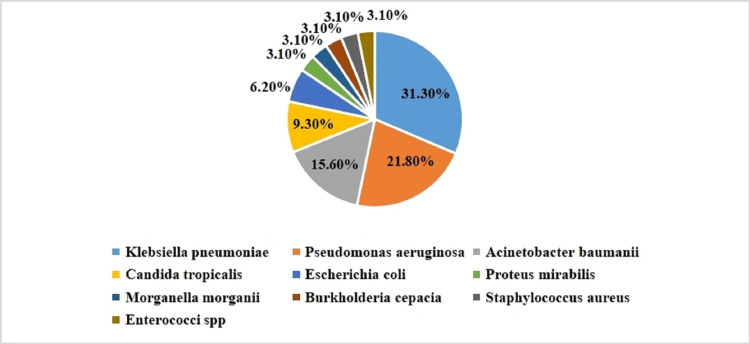
Distribution of bacterial and fungal pathogens isolated from COVID-19 patients. spp: species.

Pathogens such as *K. pneumoniae*, *E. coli*, *P. mirabilis*, *M. morganii*, *P. aeruginosa*, *B. cepacia*, *A. baumanii*, *S. aureus*, *Enterococcus spp*., and *C. tropicalis* were isolated. Among them, *K. pneumoniae*, *P. aeruginosa*, and *C. tropicalis* were more abundant in the critically ill ventilation (severe) patients than in non-ICU ward patients, whereas *K. pneumoniae* was common in all three groups. In the case of the non-ICU ward group, the predominant pathogens were *K. pneumoniae*, *E. coli*, and *A. baumanii* (Figure 1).

**Figure 2:**
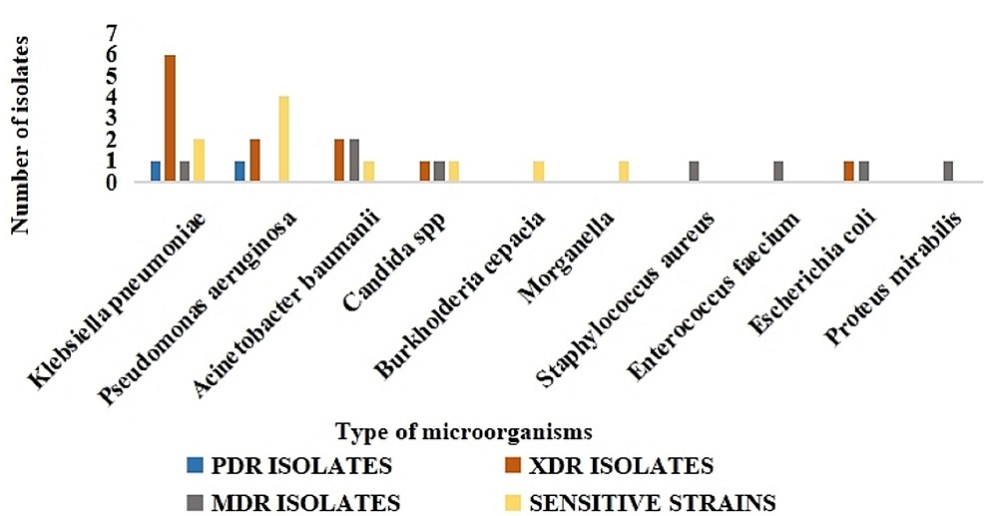
Classification of organisms according to European Centre for Disease Control (ECDC) and Centre for Disease Control Atlanta (CDC) as pan drug resistant, extremely drug resistant, multi-drug resistant, and sensitive strains.

Of the total 32 isolates, 22 (68.7%) were resistant organisms, of which two were pan drug-resistant (PDR) and 12 were extensively drug-resistant (XDR), and eight were multi-drug resistant (MDR) (Figure 2 and Table 4).

**Table 4:** Microbial profiling of different groups of long COVID patients.

Organism isolated	Number of isolates in each category of patient			Total no. of isolates
	Critical group on mechanical ventilation	Severe group on oxygen	General ward	
K. pneumoniae	5	4	1	10
E. coli	0	0	2	2
P. mirabilis	1	0	0	1
M. morganii	1	0	0	1
P. aeruginosa	5	1	1	7
B. cepacia	0	1	0	1
A. baumanii	1	1	3	5
S. aureus	0	0	1	1
E. faecalis	1	0	0	1
C. tropicalis	3	0	0	3
Total no. of isolates	17	7	8	32

**Figure 3:**
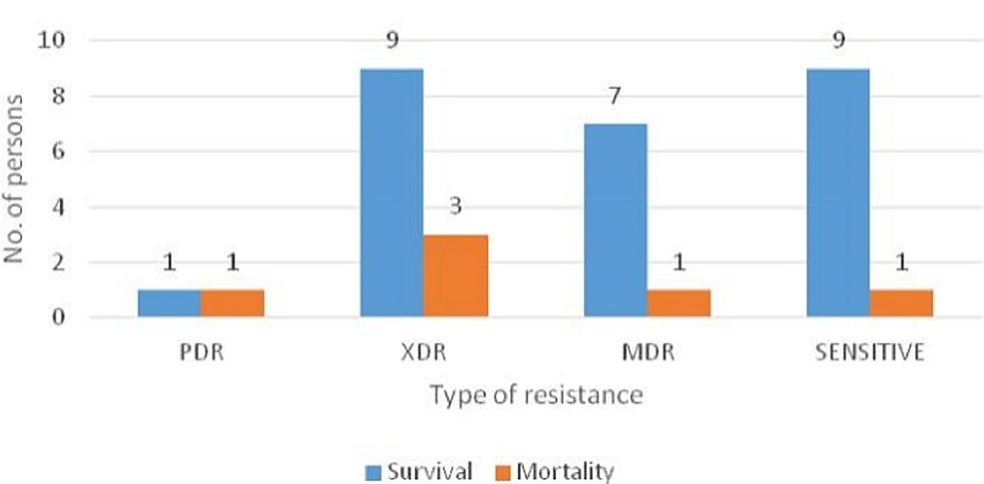
Correlation of mortality and survival with drug-resistant strains. PDR: pan drug-resistant, MDR: multi-drug-resistant, XDR: extensively drug-resistant.

**Figure 4:**
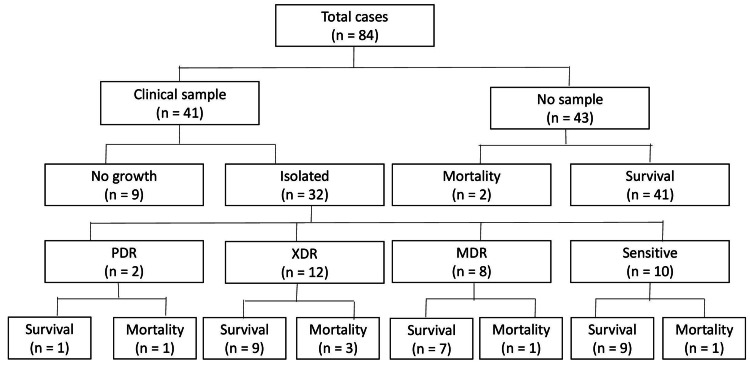
Flow chart of clinical outcome of long COVID pneumonia cases. Flow chart showing out come of long COVID pneumonia cases in the ward and ICU depicting two deaths among culture negative and six deaths among the culture positive cases, respectively. PDR: pan drug-resistant, XDR: extensively drug resistant, MDR: multi-drug resistance.

Figures 3-4 show the correlation between survival and resistance, which shows a good prognosis among sensitive strains, whereas the prognosis worsens as the resistance increases from MDR to XDR rather than PDR.

Correlation of isolated pathogens with clinical outcome

Out of 32 patients, 10 (31%) harbored sensitive organisms that included *P. aeruginosa*, *K. pneumoniae*, *M. morganii*, and *B. cepacia* in the Gram-negative group, and *C. tropicalis*.

Out of 22 (69%) patients, we witnessed PDR, XDR, and MDR patterns in 18 patients that included *K. pneumoniae*, *P. aeruginosa*, *A. baumanii*, *E. coli*, and one each of Gram-positive *S. aureus*, *E. faecalis*, and two of *C. tropicalis* (Table 5). Pathogens like *K. pneumoniae*, *P. aeruginosa*, and *A. baumanii* harbor resistance strains and are associated with a maximum number of deaths (5).

**Table 5:** Resistance (n = 5) pattern of the isolates-and correlation with mortality (n = 6). Total n = 22 resistant out of n = 32 culture isolates. PDR: pan drug-resistant, XDR: extensively drug-resistant, MDR: multi-drug-resistant.

Sl.no.	Name of organism	Death among total isolates	PDR isolates	XDR isolates	MDR isolates	Sensitive strains
1	K. pneumoniae	2/10	0/1	2/6	0/1	0/2
2	P. aeruginosa	2/7	1/1	0/2	0	1/4
3	A. baumanii	1/5	0	1/2	0/2	0/ 1
4	C. tropicalis	0/3	-	0/1	0/1	0/1
5	B. cepacia	0/1	0	0	0	0/1
6	M. morganii	0/1	0	0	0	0/1
7	S. aureus	0/1	0	0	0/1	0
8	E. faecalis	1/1	0	0	1/1	0
9	E. coli	0/2	0	0/1	0/1	0
10	P. mirabilis	0/1	0	0	0/1	0
Total		6/32	1/2	3/12	1/8	1/10

Table 6 depicts the correlation of mortality with resistance pattern, which shows that, among the six deaths, four occurred in those cases where Gram-negative bacteria were isolated and one where a Gram-positive organism was isolated, and the majority of these organisms were drug-resistant. Most of the deaths occurred in critically ill patients who were on invasive mechanical ventilators.

**Table 6:** Mortality results from the site of the sample of body fluid according to the pathogen and its resistance pattern. PDR: pan drug-resistant, MDR: multi-drug-resistant, XDR: extensively drug-resistant.

Sl. No.	Organisms isolated	Type of resistance	Sample from which isolated	No. of deaths
1	K. pneumoniae	XDR	Sputum	2
2	P. aeruginosa	PDR	Sputum	1
		Sensitive	Endotracheal secretion	1
3	E. faecalis	MDR	Blood	1
4	A. baumanii	XDR	Blood	1

## Discussion

Long COVID is a constellation of symptoms of multisystem involvement that consists of fatigue, dyspnea, cough, cognitive mental disorders, headache, myalgia, anosmia, ageusia, joint pain, loose motion, etc. The symptoms can persist beyond four weeks after COVID infection or can reappear after discharge from the hospital, although both groups of cases are RTPCR negative.

Out of all, 47.6% of patients required ICU admission and 26.2% required mechanical ventilation, which is similar to the findings observed in Colombia, where the proportion of patients requiring ICU admission was 28.8% and those requiring mechanical ventilation was 26.6% [8].

Among the total of 40 patients admitted to the ICU, 22 (55%) required mechanical ventilation. In a study conducted by Contour et al., which analyzed only ICU patients, 90% of them required mechanical ventilation [9].

Based on all the available retrospective data analysis, we concluded that 30 patients (35.7%) had bacterial or fungal co-infections, although this proportion would have been greater if samples from all patients had been sent. Studies in different parts of the world have estimated that among patients suffering from COVID-19, 1-50% of the patients have bacteria or fungus isolated from their clinical samples [8]. In a study done in India, 3.6% of patients had secondary infections [10]. This can be explained by the differences in study criteria and diagnostic tests used.

Out of the 29 samples from the ICU (both on mechanical ventilation and oxygen therapy), bacteria or fungus were isolated from 24 samples (82.7%), whereas this figure varied from 8.5% to 14% in different studies [11]. The proportion of patients in the ward whose samples showed culture positivity was 66.6% (8 samples out of 12 were culture positive), whereas it was 4% in other studies [11]. More than 70% of patients in our study were male, which could be due to outdoor and community exposure because of occupation or socialization. The majority of patients belong to the mean adult age group. This could be due to the prevalence of comorbidities in these groups of patients. Many patients have one of the comorbid illnesses, among which diabetes and hypertension are more common. These findings support the fact that the severity of COVID and post-COVID complications is more common in patients with an existing comorbid illness. Respiratory symptoms like cough, dyspnea, and chest pain were more common, while extrapulmonary presentations of fever and fatigue were also witnessed.

Radiological features indicated tissue damage. Regardless of the COVID severity, lung fibrosis was diagnosed in patients after six months of COVID positivity and there was reduced lung diffusion capacity, which was substantiated by radiological evidence of pulmonary shadows [12]. Radiological abnormalities observed were ground-glass opacity, seen in around 59% of patients, and fibrosis in 4% of cases. Consolidation was seen in 43% of patients due to pneumonia.

Elevated biomarkers (D-dimer and CRP) in the serious and critical population suggest that biomarkers may have some role in the pathogenesis of long COVID syndromes [13].

The elevated D-dimer level may be a clinical presentation of severe viral infection. Venous thromboembolism, which could be the indirect manifestation of a cytokine-induced inflammatory episode, results in an imbalance of coagulation and fibrinolysis in the alveoli, causing an increased level of D-dimer. Similarly, in the study by Mandal et al., increased D-dimer and CRP levels were more common in COVID survivors than in fully recovered cases [14]. In our study, the increased D-dimer may be the manifestation of post-COVID-induced persistent viral inflammation. Elevated CRP in our study is also a consequence of inflammation or tissue damage. The liver and renal function dysfunction in our patients can be explained by cytokine-induced outcomes due to sepsis [15]. Blood dyscrasias like leukocytosis and leucopenia were observed in severe and critical patients. Increased leukocytosis (neutrophilic) indicates a host response to inflammatory episodes. It could also be due to the aftermath of corticosteroid therapy or due to superinfection in the immune-compromised lung following corticosteroid administration of broad-spectrum antibiotic therapy. Secondary infection in the structurally damaged lung as a sequelae of complications of post-COVID fibrosis could also be a cause of leukocytosis [16].

Of the 32 isolates, 29 bacteria and 3 fungi were isolated. Gram-negative pathogens constituted 84.3% of the total isolates, which is in agreement with the findings of Vijay et al., in which the proportion of patients developing Gram-negative infections was 78% [10]. Co-infection with Gram-negative organisms cannot be attributed to COVID-19 alone as it is similar to the types of pathogens frequently associated with hospital-acquired pneumonia or ICU/hospital-acquired pneumonia as a complication of ICU care [17].

Of the total isolates, *K. pneumoniae* constituted 31.3% and *P. aeruginosa* 21.8%, followed by *A. baumanii* 15.6%, *E. coli* 6.2%, *P. mirabilis* 3.1%, *M. morganii* 3.1%, *B. cepacia* 3.1%, *S. aureus* 3.1%, and *E. faecalis* 3.1%. In another study, organisms isolated from COVID-19 infected patients were Klebsiella spp., *S. aureus*, Enterobacter spp., and *P. aeruginosa* (32%, 24%, 12%, and 10.7%, respectively) [8]. The findings of Contou et al. illustrate that the most common bacterial species were *S. aureus* (31%), *Haemophilus influenzae* (22%), *Streptococcus pneumoniae* (19%), Enterobacteriaceae (16%), *P. aeruginosa* (6%), *Moraxella catarrhalis* (3%), and *A. baumanii* (3%) [9].

In our study, all the three isolates of fungi (9.6%) were yeast (*C. tropicalis*), whereas the yeast isolated by Vijay et al. was 21.4%. The same study also showed co-infections with *Aspergillus flavus*, *Aspergillus fumigatus*, and *Candida glabrata* [10].

Out of 32, 23 positive samples (71.8%) were isolated from respiratory sites (endotracheal aspirates and sputum), whereas, in the study by Vijaya et al., most of the isolates (85%) were from blood (44.1%) and from respiratory secretions (35.1%). Secondary bacterial infections in COVID-19 patients are a stronger predictor of death compared to influenza patients [17].

The major proportions of the MDR strains were *K. pneumoniae* (36.4%), *A. baumanii* (18.2%), and *P. aeruginosa* (13.6%). In a study done in India, XDR *K. pneumoniae* and *A. baumanii* constituted almost 50% of the isolates, which is comparable to our study, wherein the total XDR isolates of *K. pneumoniae* and *A. baumanii* were 12 (54.5%) [10].

*K. pneumoniae* was the major resistant pathogen isolated among the Gram-negative isolates, followed by *A. baumanii*, *P. aeruginosa*, and *E. coli*. None of the *C. tropicalis* isolates were in the PDR group. *K. pneumoniae* is a common cause of drug-resistant hospital-acquired pneumonia in long-term facilities. This resistance in *K. pneumoniae* could be due to its plasmid-encoded extended-spectrum beta-lactamases (ESBLs), resulting in increased resistance to third and fourth-generation cephalosporins, aminoglycosides, tetracycline, co-trimoxazole, and fluoroquinolones. It can also cause community-acquired pneumonia due to its hypervirulent strain in healthy people. 

*P. aeruginosa* is a major cause of ventilator-associated pneumonia, with predisposing factors being infection, immunosuppression due to neutropenia, diabetes, and broad-spectrum antibiotic therapy. *A. baumanii* can also colonize the airway and can be responsible for nosocomial infections in ICUs with similar risk factors. Infections by *M. morganii* resemble Proteus spp infections in their clinical manifestations but are seen in people in long-term care facilities. Among hospitalized patients, it is an uncommon cause. *Burkholderia cepacia* can colonize the airways during broad-spectrum antibiotic administration and can cause ventilator-associated pneumonia and catheter-associated infection.

Methicillin-resistant *S. aureus* (MRSA) is responsible for ventilator-associated pneumonia and bacteremia and can attack immunocompromised patients and those with indwelling catheters. It can also cause community-acquired infections and severe diseases in immune-competent people [17]. 

Candida infections can be responsible for bloodstream or pulmonary infections as an opportunistic pathogen in ICU setups. Mortality was mostly observed in resistant strains, and these were mostly isolated from pulmonary secretions that indicate the severity of pneumonia as an underlying cause of mortality. Candidaemia in our case indicates an underlying immune-compromised state due to past COVID illness, comorbidities, and a longer stay in ICU.

In our study, a 91% cure rate and discharge among the critical cases were significant (p<0.00). This could be attributed to optimized ventilator management and proper antibiotic stewardship even in the face of adverse factors like increased biomarkers of inflammation, comorbid illness, renal impairment, and immunocompromised states (due to prior steroid administration and prior exposure to broad-spectrum antibiotics) in our group of long COVID cases.

The number of patients enrolled in the study was not adequate to substantiate the conclusion because of the declining trend of COVID cases within the period, which was the major limitation of the study.

## Conclusions

Eighty-four patients have been admitted as long COVID pneumonia cases, which had a 91% survival rate after target or specific antimicrobial therapy despite having the comorbid illness and raised biomarkers. *K. pneumoniae*, *A. baumanii*, *P. aeruginosa*, *E. coli*, *P. mirabilis* as Gram-negative, MRSA, *E. faecalis* as Gram-positive, and *C. tropicalis* as fungus were resistant isolates from pulmonary lesions, seen mostly among the critical cases. This survival advantage could be due to the better threshold of ICU care, judicious antimicrobial administration, and keeping antimicrobial stewardship as a priority. Death was noticed among ICU patients, those suffering from pneumonia, and those harbouring multi-drug-resistant Gram-negative organisms.
